# What Differentiates Poor- and Good-Outcome Psychotherapy? A Statistical-Mechanics-Inspired Approach to Psychotherapy Research, Part Two: Network Analyses

**DOI:** 10.3389/fpsyg.2020.00788

**Published:** 2020-05-20

**Authors:** Giulio de Felice, Alessandro Giuliani, Omar C. G. Gelo, Erhard Mergenthaler, Melissa M. De Smet, Reitske Meganck, Giulia Paoloni, Silvia Andreassi, Guenter K. Schiepek, Andrea Scozzari, Franco F. Orsucci

**Affiliations:** ^1^Department of Dynamic and Clinical Psychology, Sapienza University of Rome, Rome, Italy; ^2^Department of Psychology, NCIUL University, London, United Kingdom; ^3^Istituto Superiore di Sanità (ISS), Rome, Italy; ^4^Department of History, Society and Human Studies, University of Salento, Lecce, Italy; ^5^Faculty of Psychotherapy Science, Sigmund Freud University, Vienna, Austria; ^6^Clinic of Psychosomatic Medicine and Psychotherapy, Ulm University, Ulm, Germany; ^7^Department of Psychoanalysis and Clinical Consulting, Ghent University, Ghent, Belgium; ^8^Paracelsus Medical University, Salzburg, Austria; ^9^Faculty of Economics, Niccolò Cusano University, Rome, Italy; ^10^Psychoanalysis Unit, UCL University of London, London, United Kingdom

**Keywords:** psychotherapy, complex systems, statistical mechanics, process of change, non-linear dynamics

## Abstract

Statistical mechanics is the field of physics focusing on the prediction of the behavior of a given system by means of statistical properties of ensembles of its microscopic elements. The authors examined the possibility of applying such an approach to psychotherapy research with the aim of investigating (a) the possibility of predicting good and poor outcomes of psychotherapy on the sole basis of the correlation pattern among their descriptors and (b) the analogies and differences between the processes of good- and poor-outcome cases. This work extends the results reported in a previous paper and is based on higher-order statistics stemming from a complex network approach. Four good-outcome and four poor-outcome brief psychotherapies were recorded, and transcripts of the sessions were coded according to Mergenthaler’s Therapeutic Cycle Model (TCM), i.e., in terms of abstract language, positive emotional language, and negative emotional language. The relative frequencies of the three vocabularies in each word-block of 150 words were investigated and compared in order to understand similarities and peculiarities between poor-outcome and good-outcome cases. Network analyses were performed by means of a cluster analysis over the sequence of TCM categories. The network analyses revealed that the linguistic patterns of the four good-outcome and four poor-outcome cases were grounded on a very similar dynamic process substantially dependent on the relative frequency of the states in which the transition started and ended (“random-walk-like behavior”, adjusted *R*^2^ = 0.729, *p* < 0.001). Furthermore, the psychotherapy processes revealed statistically significant changes in the relative occurrence of visited states between the beginning and the end of therapy, thus pointing to the non-stationarity of the analyzed processes. The present study showed not only how to quantitatively describe psychotherapy as a network, but also found out the main principles on which its evolution is based. The mind, from a linguistic perspective, seems to work-through psychotherapy sessions by passing from the most adjacent states and the most occurring ones. This finding can represent a fertile ground to rethink pivotal clinical concepts such as the timing of an interpretation or a comment, the clinical issue to address within a given session, and the general task of a psychotherapist: from someone who delivers a given technique toward a consultant promoting the flexibility of the clinical field and, thus, of the patient’s mind.

## Introduction

In the history of science, many efforts have been made to identify coarse-grained descriptors that can explain the behavior of complex systems composed of several different elements. Statistical mechanics describes physical phenomena in terms of the stochastic (random) behavior of a large numbers of components, such as atoms or molecules, focusing on the distribution of energy among these components. As such, statistical mechanics provides exact methods to connect thermodynamic quantities (e.g., pressure, volume, and temperature) to microscopic behavior (e.g., the behavior of a large number of atoms of a given gas). The importance of statistical mechanics resides in the development of coarse-grained statistical descriptors able to catch the essential features of a system regardless of its microscopic behavior. A crucial concept in this approach is the notion of “ensemble”: an abstraction consisting of a large number of virtual copies (sometimes “infinitely many”) of a system, considered all at once, each of which represents a possible state that the real system might occupy in a given instant of time (Hill, 1986). In other words, it is a collection of a large number of systems that are macroscopically identical (e.g., the isothermal–isobaric ensemble groups together the processes of the systems in which temperature and pressure are constant). Hence, statistical mechanics investigates the possibility to extract few relevant “macroscopic” features of a physical system described as “average quantities.” In so doing, the discipline has always emphasized the importance of examining the instances in which complex processes undergo a drastic simplification as it allows for the characterization of the system as a whole in terms of few “order parameters” (here, avoiding the penumbra of associations around this technical word used in the domain of statistical mechanics, we will use the term “macro-parameters”).

In the present study, we rely on the rationale of statistical mechanics, fostering its application to psychotherapy research in order to examine the possibility to reduce the complexity of the psychotherapeutic system into few coarse-grained empirical macro-parameters. From a clinical perspective, this effort of abstraction constituted the main objective of the entire work by Wilfred R. Bion, who is, according to the mainstream, the most important psychoanalyst of the modern era. In “Elements of Psychoanalysis”, the author abstracts and describes the functioning of the three main elements of the mind, namely, the oscillation between the schizo-paranoid and depressive position (PS-D), the container–contained interaction (♀♂), and the linkages L–H–K (Bion, 1984). From an empirical perspective, the need for reducing the complexity of the psychotherapeutic system into few coarse-grained macro-parameters is justified by the continuously increasing number of identified single- and multiple-outcome predictors (de Felice et al., 2019a). In a recent systematic review on the outcome of cognitive–behavioral therapy for eating disorders, for instance, Linardon et al. (2017) found 6 mediators, 13 moderators, and 20 predictors while considering only patient characteristics (i.e., excluding any relational or therapist-related variables); also, no other forms of therapies or diagnoses were included in the review.

In the field of biology, many studies (e.g., Jolliffe and Cadima, 2016; Mojtahedi et al., 2016; Pagani et al., 2016; Giuliani et al., 2018) demonstrated the substantial usefulness of looking at biological systems from the perspective of statistical mechanics. In particular, it allowed one to reduce the hyper-complexity underlying a given phenomenon by focusing on the mutual correlations among system descriptors. This lens for observing the complexity underlying a given phenomenon, in biology, has been called the “middle-out” approach, since it focuses on the correlation among intervening variables (i.e., not “microscopic” raw variables, but macro-parameters calculated over their interactions), and lies between the hyper-complexity and hyper-simplification of the phenomenon under consideration. This approach has enabled researchers to describe complex biological systems through few macro-parameters mainly linked to changes in the degree of correlation of the system at hand (e.g., Laughlin et al., 2000; Giuliani et al., 2014).

Along these lines of thought, highlighting the importance of abstracting few macro-parameters to study the complexity of a given system, in the psychotherapy research literature, Schiepek and Strunk (2010) formulated an empirical dynamic descriptor that can predict therapeutic change and showed to be linked with good outcome. The descriptor, called “dynamic complexity” (indicated with “C”), was obtained by the multiplication of the distribution (D) and fluctuation (F) of a given variable (C = D × F; for a detailed description, see Schiepek and Strunk, 2010); it can be used as a measure of the complexity of a system. Specifically, a peak of dynamic complexity has been found to precede therapeutic change, consistent with the statistical mechanics’ theory of “tipping points” preceding critical transitions (Scheffer et al., 2012). The clinical counterpart of a “tipping point”, and thus “a peak of dynamic complexity”, can be the observation of something new occurring in the patient’s in-session narratives (e.g., an insight) or in some of his/her behaviors outside the clinical room (e.g., see photographs of childhood or be interested in previously insignificant things) (e.g., Gumz et al., 2010, 2012). Other similar applications based on dynamic systems are described in previous works (see Guastello et al., 2009; Pincus and Guastello, 2013; Gelo and Salvatore, 2016). Moreover, there are empirical studies that described psychotherapeutic processes in terms of stable dynamic patterns are worth mentioning (see Gelo et al., 2008; Tschacher and Ramseyer, 2009). In these works, initial attempts have been made to investigate the evolution of psychotherapy using coarse-grained empirical indices. However, despite these initial efforts, a fully consistent empirical proof of the possibility to predict the evolution of psychotherapy by means of quantitative macro-parameters of order, variability, and complexity has never been obtained. In this study, we aim to define a self-consistent procedure to compare psychotherapies with different orientations and different outcomes considering only the correlation pattern among their macro-parameters. Specifically, the primary and secondary research objective guiding the research project are (a) the possibility of predicting good- and poor-outcome psychotherapies on the sole basis of the correlation pattern among their macro-parameters, and (b) the investigation, in terms of those correlation patterns, of analogies and differences between the processes of good- and poor-outcome cases.

The first two steps of this investigation were examined in a previous work using the same dataset (see de Felice et al., 2019b); this study led to the following results:

1. By means of “static analyses” we were able to highlight significant differences between good- and poor-outcome cases concerning their latent correlation structures. The most evident difference was linked to the patients’ use of abstract language, interpreted very positively by the therapists of poor-outcome cases and very negatively by the therapists of good-outcome cases. This observation was associated with the use of positive and negative emotional languages inversely proportional to abstract language in poor-outcome patients. Overall, this configuration was interpreted as a dynamic of “rationalization” occurring in poor-outcome patients only.
2. Regarding the “dynamic analyses”, the results showed the possibility to describe the psychotherapy process, independently from the theoretical approach, with two quantitative dimensions (macro-parameters), namely, order-variability and elementary-complex. These two macro-parameters were statistically significant in describing the trajectory of each psychotherapy of the sample and, in so doing, supported the application of a statistical-mechanics approach to psychotherapy research.

Complementing these results, the present work investigates the analogies and differences of the linguistic networks of good- and poor-outcome cases using a network analysis approach.

## Materials and Methods

### Sample

The sample was drawn from the York Depression Study I, a randomized clinical trial on the efficacy of brief experiential therapy [client-centered therapy (CCT) and emotion-focused therapy (EFT)] for depression (e.g., Watson et al., 1998). The York Depression Study I originally involved 17 CCT and 17 EFT treatments. For the present study, a subsample, the six best-outcome cases (CCT = 3; EFT = 3) and the six worst-outcome cases (CCT = 3; EFT = 3), was selected. The selection was based on the Reliable Change Index (i.e., RCI; Jacobson and Truax, 1991) of the Beck Depression Inventory (BDI; Beck et al., 1961, 1988). Then, four cases (1 = EFT; 3 = CCT) were excluded due to some missing sessions. The eventual sample, therefore, comprised eight cases: four with a good outcome (1 = CCT and 3 = EFT) and four with a poor outcome (2 = CCT and 2 = EFT) (Table 1). For more details on the sample, see Mendes et al. (2010).

**TABLE 1:** Descriptive statistics of the sample.

Case number	Client acronym	Treatment	BDI pre–post improvement	Outcome
1	Primo	EFT	1	Poor
2	Secondolo	CCT	6	Poor
3	Terzio	CCT	4	Poor
4	George	EFT	2	Poor
5	Jan	EFT	25	Good
6	Margareth	CCT	12	Good
7	Lisa	EFT	22	Good
8	Sarah	EFT	31	Good

*EFT, Emotion-focused therapy; CCT, client-centered therapy; BDI, Beck Depression Inventory.*

### Patients

The patients were one man and seven women with a mean age of 37.08 years (SD = 12.43); all met the criteria for major depressive disorder (MDD) as defined by the Structured Clinical Interview for DSM-III-R (SCID; Spitzer et al., 1989).

### Therapists

The therapists were seven women and one man with an average of approximatively 5.5 years (SD = 1.7) of therapeutic experience and 24 weeks of training in experiential psychotherapy (Greenberg et al., 1993, 1994). Only one patient was assigned to each therapist, resulting in eight different therapeutic dyads. All therapists were monitored for adherence using video recordings of the therapy sessions and engaged in weekly supervisions during the period of the investigation.

### Treatments

Client-centered therapy emphasizes the use of empathy, positive regards, and congruence (see, for instance, Rogers, 1951; Greenberg et al., 1994). EFT integrates CCT with “process-directive gestalt and experiential interventions” for the resolution of dysfunctional cognitive–affective processing (Watson et al., 1998, p. 210). The treatment length was between 15 and 20 sessions (*M* = 17.62, SD = 1.38), for a total of 141 sessions.

### Measures

The semantic production of the eight brief psychotherapies was coded according to Mergenthaler’s Therapeutic Cycle Model (TCM; Mergenthaler, 1996, 2008, 2011). This is a computer-assisted deductive content analytic tool that breaks the transcript down into chunks of 150 word-blocks and subsequently analyzes these word-blocks according to three different categories (called “dictionaries”): (a) positive emotional tone (POS), (b) negative emotional tone (NEG), and (c) abstraction (AB). The first two contain adjectives, verbs, or adverbs with a positive or negative valence (e.g., happy, sad; agree, disagree; hug, abandon; incredible, astonished). The third contains abstract words (e.g., year, hour, accident, soul, and wedding). All sessions were transcribed according to the TCM international standards (Mergenthaler and Stinson, 1992). The TCM automatically assesses the relative frequency of the three dictionaries for each word-block.

In short, the dataset included six variables (statistical descriptors) as columns – abstract, positive, and negative language pertaining to patient and therapist of each therapeutic dyad – and the word-blocks in temporal order as rows (statistical units).

### General Methodological Considerations

The limited sample size makes our investigation more similar to a “feasibility study” with a methodological aim than to a classical hypothesis testing approach. At this stage of development, we wish to give a proof of concept of the consistency, stability, and interpretability of the results grounded on the application of such a new methodological path. In so doing, we controlled for the presence of evident biases in the analyses. Concerning the experimental sample selection, this check was based on the inclusion of patients with similar age and psychotherapists with a comparable clinical experience. The reliability of the dynamical profiles (Markov Transition Matrices) stemmed from both the adequate length of the analyzed series (each statistical unit is a 150 word-block pertaining to an entire brief psychotherapy with an average of 17 sessions) and the elimination of scarcely populated clusters (states). It is worth noting that, in order to promote the passage from the search of a “proof-of-concept” to a fully operative investigation, it will be necessary to collect a much higher number of subjects.

### Data Analysis

In order to transform, visualize, and investigate the psychotherapeutic process as a network, we used a symbolic dynamics approach: we considered each psychotherapy as a discrete time series consisting of different states that are progressively visited by the system (i.e., psychotherapeutic relationship). The states are generated by a data-driven clustering technique based on the co-occurrence of patterns of elementary symbols. Cluster analysis is routinely used to code time series as sequences of discrete states in which each state corresponds to a cluster (Kitchens, 2012). This transformation allows one to develop a reliable symbolic dynamic (Karpen et al., 1993), in this case, a time series of clusters representing the evolution of the patient’s linguistic behavior over time (Appendix 1).

Each configuration of the linguistic variables (i.e., POS, NEG, and AB) in a given time point can be seen as a specific state of the system or node of its network and represents the position of the system in a multidimensional space. The configurations that recur over time pertain to the same cluster that, in turn, can be considered as “quasi-attractors” (i.e., a more stable state) of the psychotherapeutic system (Karpen et al., 1993; Graben and Hutt, 2015) (Appendix 1). The transitions of the system across such states is represented by a network having clusters as nodes (i.e., states of the system) and the frequency of the transition between one state (*i*) and another (*j*) as edges. In order to spot potential differences between good- and poor-outcome dynamics, the transition probabilities between different clusters are studied through Markov matrices. The rows of Markov Transition Matrix (MTM) represent the conditional probability of going from state *i* (row) to state *j* (column) in a single step. The matrix corresponds to a phase space diagram having as rows the *Xt* values and as columns the *Xt* + 1 values (i.e., the states of the system at time *t* and *t* + 1). The values within each cell, *Tmij*, represent the probability of going from state *i* to *j* in a single step; thus, they correspond to the observed conditional probabilities at subsequent points in time: *P*[*I*(*t*)]| *J*(*t*−1) (Feller, 1968). MTMs offer a way to generate the characteristic network of good- and poor-outcome cases while at the same time presenting each therapist’s and patient’s individual network.

Hence, the distribution of cluster transitions was subsequently analyzed for both good- and poor-outcome cases as well as for both the therapists and patients. The comparison between different dynamics was accomplished by using both a direct statistic (Pearson correlation between pairs of MTMs) and a model-mediated measure (multivariate regression model testing the relative weights of the distance between state *i* and *j*, and their relative frequencies to predict the transition probabilities). The Pearson correlations between networks of poor- and good-outcome cases can be seen as a measure of the stability and accuracy of the clusterization. The higher the Pearson correlations, the more the clusterization was able to catch the main information in the eight psychotherapeutic processes.

On the other hand, the model-mediated measures (multivariate models, Table 5) are based on the significance of two regressors (see the next section for details). The model gives rise to two numerical indices representing the normalized coefficients of the two regressors for both patients and therapists (β values, Table 5). The sensitivity of such bi-dimensional description in grasping the main information of both the patients and therapists’ networks can be observed in the proportion of variance explained by the model (adjusted *R*^2^, 0.75 for patients, 0.68 for therapists, Table 5): such level of accuracy is fully satisfactory.

Since it is easier to understand and visualize a procedure through direct application, a more detailed description of the method is provided in the section hereafter.

## Results

In order to study the dynamic interaction between patients and therapists and the potential difference in poor- and good-outcome cases, we applied symbolic dynamics, a mathematical procedure widely used to discretize continuous variables, revealing their temporal occurrences. Therefore, we studied the best cluster solution for the linguistic data matrix of patients and therapists separately (i.e., three dictionaries, abstract language “AB”, positive emotional “POS” and negative emotional “NEG” language for patients and therapists). We took the two most broadly used clusterization algorithms, namely, K-means and Minimum Spanning Tree, into consideration. The best cluster solution, balancing quantity of information and clarity of interpretation, turned out to be K-means with eight clusters for both patients and therapists, explaining the 65% and 68% of the variance, respectively. The rationale of applying the clusterization to patients and therapists separately is that this allows one to study their dynamic interactions over time, information that would have otherwise been missed. Hence, the K-means algorithm was applied separately over the three linguistic variables of patients and therapists. This resulted in a numeric label (from one to eight) for each statistical unit (or row of the dataset), indicating the cluster the specific observation pertains to. Each number corresponds to a specific state of the system or profile of the three dictionaries, and when that specific state recurs over time, so does the number generated by the algorithm. Clinically, this could be seen as a study of invariants of the patients’ narratives (e.g., the patient’s object relations are repetitive patterns trough which he/she perceives the reality and himself/herself).

The clusters that were very scarcely populated, that is, with very few occurrences (two for the patients and three for the therapists) were considered “outliers” and consequently their statistical units were deleted from the investigation. It is worth noting that these outliers, considering the multidimensional scaling, also lay at a very far distance compared to the other clusters. This confirms the substantial consistency of the resulting phase space of the therapeutic processes (i.e., distant regions of the space are only very rarely explored by the system). Accordingly, the accepted cluster solutions were six and five clusters (or states of their systems) for patients and therapists, respectively. See Appendix 2 for the multidimensional scaling planes, clusters’ frequencies, and centroids of the patients and therapists’ phase space.

**FIGURE 1:**
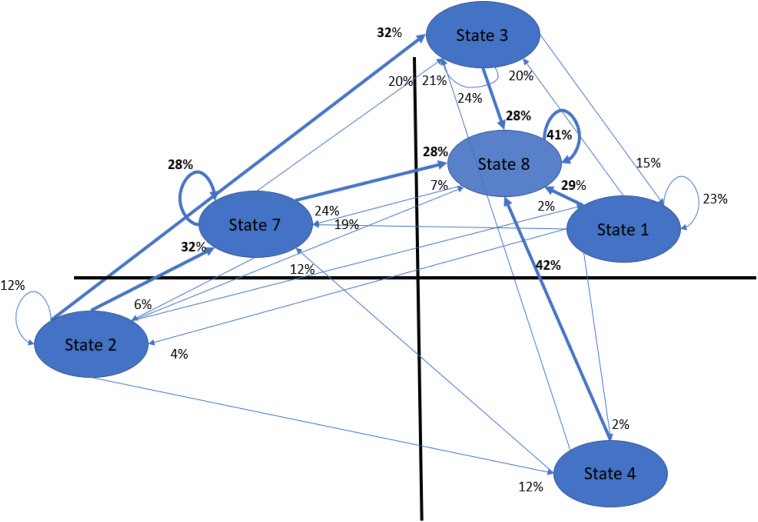
Graphic visualization of George’s MTM or George’s linguistic network. Transitions with the highest occurrence for each state are in bold. In this case, we see a clear tendency to pass through state 8 (i.e., silence, the only state in which all three dictionaries show a minus sign), which is the most frequent state with 2191 occurrences (on a total of 7388). Second ranked was cluster 3 with 1494 occurrences, followed by cluster 7 (1484), and finally, cluster 1, cluster 4, and cluster 2, with 1115, 709, and 371, occurrences, respectively (see Appendix 2, Table III). To interpret the profile of each state, see Appendix 2, Table II.

The time series of clusters generated by the K-means algorithm (i.e., eight for the patients and eight for the therapists) gave rise to first-order Markov transition matrices. Rows and columns contained the different clusters; the elements of each cell represented the normalized frequency of a direct (single step) transition from row (*i*) to column (*j*). Hence, in each cell of the matrix, there was a relative probability of passing from the state or cluster in row to that in column. We show, as an example, the transition matrix of the poor-outcome patient George (Table 2 and Figure 1).

**TABLE 2:** The number of clusters or states of the system is indicated in the rows and columns.

	George (poor outcome): Markov transition matrix							
	1	2	3	4	5	6	7	8
1	0.237	0.042	0.203	0.025			0.195	**0.297**
2	0.025	0.125	**0.325**	0.125			**0.325**	0.075
3	0.159	0.057	0.248	0.038			0.217	**0.280**
4	0.121	0.030	0.212	0.091			0.121	**0.424**
5								
6								
7	0.139	0.062	0.206	0.026			**0.289**	**0.278**
8	0.129	0.031	0.133	0.043			0.246	**0.418**

*Each cluster is composed of a peculiar configuration of the three linguistic vocabularies. Empty cells correspond to clusters that have been deleted because they are considered as “outliers” (scarcely populated and located at the extremes of the distribution). In each cell, the relative probability of passing from the state or cluster in row to that in column is indicated. The most probable transition for each state is highlighted in bold.*

The Markov matrices of eight patients and eight therapists seem to be highly correlated (average Pearson *r* = 0.78; st. dev. = 0.46 and *r* = 0.82; st. dev. = 0.10 for therapists and patients, respectively), pointing out a considerable invariance of the therapeutic dynamics. This represents a prominent proof of concept of the possibility to consider psychotherapy as a proper dynamical system advocating its investigation by using classical physically inspired methods (i.e., regardless of the peculiarities of each psychotherapeutic process, there is a large amount of shared information among these eight cases; this allows us to investigate the principles ruling this common dynamic). It is worth noting that the standard deviation of the Pearson correlation was four times higher for therapists than for patients, suggesting that while therapists attempt to “dynamize” the clinical field, the patients’ linguistic behavior is more redundant.

Subsequently, in order to shed more light on the different behaviors characterizing the dynamics of patients and therapists, we used a new combined index, here named as “DeltaCorr” (Equation 1).

Equation 1. “DeltaCorr.”

DeltaCorr(i,j)=CorrPat(i,j)-CorrTher(i,j)

[Example:

DeltaCorr(Sarah,Lisa)=CorrPat(Sarah,Lisa)

$$-CorrTher\left(Sarah^{\prime }sTherapist,Lisa^{\prime }sTherapist\right)]$$

Where CorrPat(*i*, *j*) = Pearson correlation between the *i* and *j* Markov matrix pertaining to patients. CorrTher(*i*, *j*) = Pearson correlation between the *i* and *j* Markov matrix pertaining to therapists.

Table 3 shows the means and standard deviations of DeltaCorr across poor- and good-outcome cases.

**TABLE 3:** Means and standard deviations of DeltaCorr across different outcome classes.

Variable	*N*	Mean	St. Dev.	Minimum	Maximum
**Class = poor–poor**					
CorrTher	6	0.725	0.101	0.560	0.840
CorrPat	6	0.830	0.037	0.780	0.870
**DeltaCorr**	**6**	**0.105**	**0.100**	**−0.020**	**0.270**
**Class = good–good**					
CorrTher	6	0.845	0.059	0.760	0.940
CorrPat	6	0.796	0.031	0.760	0.850
DeltaCorr	6	−0.048	0.077	−0.130	0.090

*The highest value of DeltaCorr is indicated in bold.*

The discrepancy (DeltaCorr) between the therapists and patients’ dynamics was two times higher within poor–poor correlations than in good–good correlations, and of opposite sign (0.105 vs. −0.048, respectively). Additionally, the values of DeltaCorr within the poor–poor class (i.e., outcome) were the only ones in which the 95% confidence interval (CI = 0.025 to 0.185) did not include the zero value. Hence, we can affirm the presence of a difference, although small, between the patients and therapists’ linguistic dynamics. The greater variability within the correlations of poor-outcome therapists’ Markov transition matrices can be interpreted as a bigger effort exerted by the therapists to deal with poor-outcome patients. Of course, it is impossible to conclude whether this greater variability in the behavior of poor-outcome therapists depended on a deliberately different therapeutic approach or was rather related to the difficulty of the clinical process in which they were involved. In the latter case, it could be a sign of two opposite clinical pictures: a particularly difficult patient pushing the therapist to find new and previously unexpected solutions to manage the impasse, or a therapist who is so lost in the clinical process that tries random interpretations. Whatever the case may be, the result is an empirical increase in the standard deviation of the therapists’ linguistic behaviors and a poor therapeutic outcome measured at the end of the treatment.

So far, we gained two main insights from the application of network analyses on our dataset: we observed a very high consistency of therapists and patients’ dynamics (average Pearson *r* = 0.78; st.dev. = 0.46 and *r* = 0.82; st.dev. = 0.10 for therapists and patients, respectively), and a small but significant difference in the linguistic behavior of therapists (DeltaCorr 0.105 vs. −0.048 for poor- and good-outcome cases, respectively).

In what follows, we will move from the study of patients and therapists separately to the study of psychotherapeutic *dyads*. In order to study the psychotherapeutic dyads and their clinical processes, we made use of combined symbolic dynamics produced by the interpolation of patients and therapists’ single trajectories. For instance, GeorgePat1, GeorgeTher1, GeorgePat2, GeorgeTher2.GeorgePat(*n*), GeorgeTher(*n*), where Pat(*i*) and Ther(*i*) are the states or clusters progressively visited by patient and therapist during their interaction. This procedure makes the corresponding first-order Markov transition matrix a Patient-to-Therapist sequence of discrete transitions. As an example, the poor-outcome Patient-to-Therapist interaction of George is shown below (Table 4).

**TABLE 4:** George’s patient-to-therapist symbolic dynamic.

George patient-to-therapist dynamic								
Cluster/state	1	2	3	4	5	6	7	8
1	0.066	0.246	0.016	0.131			0.361	0.18
2	0.103	0.069	0.138	0.052			0.284	0.353
3	0.014	0.264	0	0.125			0.389	0.208
4	0.133	0.084	0.12	0.12			0.229	0.313
5								
6								
7	0.084	0.113	0.122	0.084			0.303	0.294
8	0.061	0.187	0.075	0.14			0.299	0.238

*Empty cells correspond to clusters that have been deleted because they were considered as “outliers.” In each cell, the relative probability of passing from the state or cluster in row to that in column is indicated.*

Analogously to what has been observed in the case of patients and therapists’ individual trajectories, the Patient-to-Therapist symbolic dynamics also demonstrated a very high consistency (average Pearson correlation: *r* = 0.872; st. dev. = 0.038) that proves the similarity of their interacting behaviors and dynamical principles. This observation was further confirmed by the high consistency in Therapist-to-Patient symbolic dynamics [example: GeorgeTher1, GeorgePat1, GeorgeTher2, GeorgePat2.GeorgeTher(*n*), GeorgePat(*n*); average Pearson correlation: *r* = 0.848; st. dev. = 0.044] and between Patient-to-Therapist and Therapist-to-Patient dynamics (average Pearson correlation: *r* = 0.892; st. dev. = 0.032).

After demonstrating the strong similarities between the patients’ linguistic behaviors, the therapists’ linguistic behaviors and the patient-therapist dyads, we now bring the attention to the study of the principles that determine these linguistic dynamics.

Do the symbolic dynamics of patients and therapists follow a specific principle? To answer this question, we rely on two opposite modes of functioning:

### A) Distance-Dependent

The transition dynamic depends on the Euclidean distance between clusters (i.e., most of the transitions occur between neighboring clusters, only a minority take place between distant ones). In this case, the transitions depend on the distance between the cluster from which the transition starts (*i*) and the cluster in which the transition ends (*j*). This means that the transitions between the *i* and *j* states are negatively correlated with their mutual distance. The area of the phase space (i.e., the network) in which most of the transitions take place can be regarded as the system’s attractor (i.e., the most recurrent state or group of states). In the clinical practice, it could be represented by an impasse (if the attractor is dysfunctional) or a positive transference (if the attractor is functional).

### B) Dependent on the Relative Frequency of the j State

In this case, the transitions depend on the number of occurrences of the state in which the transitions end. This means that the transitions between the *i* and *j* states are positively correlated with the relative frequency of *j*. Each state can be reached by any other state regardless of distance; the system is called “ergodic.” In the clinical practice, this could be represented by a flexible and healthy patient capable of expressing himself regardless of his anxiety (functional picture), or a patient with severe thought disorders incapable of focusing his attention on a single internal state because of extreme anxiety (dysfunctional picture).

It can be useful to consider a third mode (c) that is situated in the middle of the two aforementioned modes of functioning. This consists of a system’s trajectory that depends on the relative frequencies of both the *i* and *j* states. In this case, the transitions are positively correlated with the number of occurrences of both *i* (the state from which the transition starts) and *j* (the state in which the transition ends). This means that the system is not completely ergodic because its transitions depend, even if only partially, also from the state in which the transition starts. In other words, the system’s initial position influences the next step. This has been called the “Drunkard’s walk” [i.e., random walk, first defined by Pearson (1905)] in which, on the one hand, you will never know where the drunk man will step next, yet, on the other, the possibilities are limited by how far the drunk man can widen his legs. Clinically, it is a very common relational picture: usually, a therapist waits long enough in order for the patient to be ready to accept a given comment or interpretation, that is, until the therapist believes that the patient’s mind is sufficiently “widened.” Alternatively, in the case of an out-of-time interpretation, it is common to experience a rejection by the patient, suggesting that the interpretation was too far away from his current mind’s amplitude.

Mathematically, testing the above-outlined models would correspond to exploring the fit of a multiple regression model with the Markov transitions (i.e., the value of each cell of the Markov matrix) as dependent variable (*Y*) and, as regressors (*X*s, independent variables): the distance between state *i* and *j* (*X*1, mode “a”); the relative frequency of state *j* (*X*2, mode “b”); the product of the relative frequencies of both state *i* and *j* (*X*2, mode “c”). At the end of the procedure, each trajectory is expressed as an equation like *Y* = *aX*1 + *bX*2, where *a* and *b* are normalized coefficients and represent the relative importance of the independent variables.

The multiple regression model that most significantly described the symbolic dynamics of patients and therapists is the following (Equation 2):

Equation 2. Best fitted Multiple Regression Mode.

Y (Markovmatrixlinearized)=

aX1(distancebetweenstateiandj)+bX2(compositefrequency

=relativefrequencyofstateirelative*frequencyofstatej)

where *a* and *b* are the weights of the independent variables.

The model fitted very well for both patients and therapists’ dynamics. The mode (c), as discussed above, lies between the two proposed modes of functioning (a and b), because it makes the transitions depend not only on the state in which the transition ends but also on the state from which the transition starts. We show the results of the multiple regression model applied to each subject below (Table 5):

**TABLE 5:** Results of the multiple regression model applied to each subject.

Multiple regression model							
Patients	β distance (normalized)	β composite frequency (normalized)	*R*	*R*^2^	Adjusted *R*^2^	*p*	β composite/β distance
George	−0.295	0.709	0.864	0.746	0.717	<0.0001	2.403
Primo	−0.373	0.697	0.896	0.803	0.781	<0.0001	1.869
Secondolo	−0.405	0.663	0.896	0.803	0.781	<0.0001	1.637
Terzio	−0.149	0.806	0.872	0.761	0.734	<0.0001	5.409
Jan	−0.369	0.673	0.873	0.762	0.735	<0.0001	1.823
Lisa	−0.476	0.645	0.928	0.861	0.845	<0.0001	1.355
Margareth	−0.452	0.676	0.941	0.885	0.872	<0.0001	1.495
Sarah	−0.220	0.763	0.869	0.754	0.727	<0.0001	3.468
*Mean*	−*0.343*	*0.704*	*0.892*	*0.797*	*0.774*	<*0.0001*	*2.432*

**Therapists**	**β distance (normalized)**	**β composite frequency (normalized)**	***R***	***R*^2^**	**Adjusted *R*^2^**	***p***	**β composite/β distance**

George	0.271	1.006	0.888	0.789	0.754	<0.0001	3.712
Primo	−0.036	0.827	0.845	0.714	0.667	<0.001	22.972
Secondolo	0.057	0.915	0.89	0.793	0.758	<0.0001	16.052
Terzio	−0.025	0.784	0.798	0.638	0.577	<0.002	31.36
Jan	−0.069	0.892	0.927	0.859	0.835	<0.0001	12.927
Lisa	−0.056	0.750	0.782	0.612	0.547	<0.003	13.393
Margareth	0.109	0.875	0.831	0.690	0.639	<0.001	8.027
Sarah	0.031	0.877	0.862	0.743	0.700	<0.0001	28.29
*Mean*	*0.035*	*0.866*	*0.853*	*0.730*	*0.684*	<*0.001*	*17.092*

The results for poor-outcome patient George will be discussed as an example, they are presented in the table from left to right. “β distance” is the coefficient “*a*” of equation 2 and represents the importance of the distance between state *i* (begin) and *j* (end) in explaining the variance of George’s Markov transitions. “β composite frequency” is the coefficient “*b*” of Equation 2 and represents the importance of the relative frequencies of state *i* and *j* in explaining the variance of George’s Markov transitions. “*R*” is the square root of *R*^2^ and is the correlation between the observed and predicted values of the dependent variable. “*R*^2^” is the proportion of variance in the dependent variable (*Y*) that can be explained by the independent variables (*X*1, *X*2); it does not reflect the extent to which any particular independent variable is associated with the dependent variable. “Adjusted *R*^2^” is an adjustment of the *R*^2^ that penalizes the addition of extraneous predictors to the model. Adjusted *R*^2^ is computed using the formula 1−(1−*R*^2^)((*N*−1)/(*N*−*k*−1)), where *k* is the number of predictors. The “*p* value” represents the statistical significance of the model. “β composite/β distance” is the value resulting from dividing the two β coefficients. It represents the proportional importance of the model’s independent variables. In the case of George, the composite frequency is 2.4 times more important than distance in describing the Markov transitions.

As we can read from Table 5, the model explains the data variance for both therapists and patients very well (average *R*^2^ = 0.763; average adjusted *R*^2^ = 0.729, corresponding to 76% and 73% of variance explained). The high predictive value of the model demonstrates that the transition dynamics depended on the distance and relative frequencies of both state *i* (state from which the transition starts) and *j* (state in which the transition ends). Therefore, as an answer to the question on the nature of transition dynamics (i.e., do the symbolic dynamics of patients and therapists follow a specific principle?), we can state that there were no significant differences between good- and poor-outcome patients. The transition dynamics of both therapists and patients followed a specific trend, and for the most part, it depended on the composite frequency (the product between relative frequency of state *i* and *j*). Precisely, the composite frequency weighs 2.4 and 17 times more than the distance for patients and therapists, respectively. This difference is mainly explained by their β distance coefficients: the therapists’ transitions depended approximately 10 times less on distance than those of patients (ratio between absolute values of their β coefficients = 0.343/0.035 = 9.8). This result is in complete accordance with the greater variability of the correlations of poor-outcome therapists’ Markov transition matrices, which we interpreted as a bigger effort performed by the therapists in dealing with the poor-outcome patients (Table 3). These findings suggest that the behavior of poor-outcome therapists was more dynamic and unconstrained or, in other words, showed less dependence on the distances between states.

After having clarified the nature of the transition dynamics for both patients and therapists, we now present the analyses of the possible difference in the number of occurrences (i.e., frequencies) of the states at the beginning and end of therapy. In order to do so, we used odds ratio (chi-square statistics). An odds ratio (OR) is usually a measure of association between an exposure and an outcome. The OR represents the odds that an outcome (in our case a difference in the number of occurrences of a given state) will occur given a particular exposure (the distribution of states of that specific symbolic dynamic), compared to the odds of the outcome occurring in the absence of that exposure. In the present study, we checked for the presence of significant differences in the occurrences of states between the first part (the first 33% of observations) and the third part (the last 33%) of each psychotherapy. The results are shown below (Table 6).

**TABLE 6:** Results of odds ratios between the first and third part of each psychotherapy.

Odds ratios					
Subject	Outcome	State/cluster	First part	Third part	*p* value
Secondolo (Therapist)	Poor	1	13/322	2/322	*p* = 0.014
Terzio (Therapist)	Poor	2	53/245	78/244	*p* = 0.050
Terzio (Therapist)	Poor	7	91/245	53/244	*p* = 0.005
Lisa (Patient)	Good	8	100/257	61/255	p = 0.008
Margareth (Patient)	Good	1	47/413	69/413	*p* = 0.056
Margareth (Therapist)	Good	1	12/407	4/406	*p* = 0.059
Margareth (Therapist)	Good	4	68/407	44/406	*p* = 0.035

*For the sake of simplicity, we only show the results with a significant p value.*

We observed some significant changes in the linguistic behavior between the beginning and end of therapy for two poor-outcome therapists (the psychotherapists of Secondolo and Terzio), two good-outcome patients (Lisa and Margareth), and one good-outcome therapist (the psychotherapist of Margareth). The results corroborate the hypothesis that “poor-outcome” therapists try more often to “dynamize” their psychotherapeutic fields compared to their “good-outcome” colleagues, thereby resulting in an increased linguistic variability. Specifically, the therapist of Secondolo reduced the state with a high use of negative emotional language (cluster 1, Appendix 2, Table II). The therapist of Terzio increased the state with high abstract language (cluster 2, Appendix 2, Table II) and decreased the state with all the three dictionaries showing a minus sign (silence, cluster 7, Appendix 2, Table II).

On the patients’ side, on the contrary, it seems that the good-outcome cases are those patients showing significant changes. In particular, Lisa decreased the state with all the three dictionaries showing a minus sign (silence, cluster 8, Appendix 2, Table II). Margareth, on the other hand, increased the state with a positive sign for positive and abstract language (cluster 1, Appendix 2, Table II), while her therapist (the only good-outcome therapist showing significant changes) decreased the use of the state that was characterized by more negative emotional language (clusters 1 and 4, Appendix 2, Table II).

## Conclusion

Answering the central aim of our study, we found that the application of a statistical-mechanics-inspired approach to psychotherapy research indeed allowed us to abstract the main macro-parameters of the eight psychotherapies of our sample and to investigate the analogies and differences in the linguistic networks of good- and poor-outcome cases. We gained two main insights from the network analyses applied on our dataset:

a) A significantly greater variability in the linguistic behavior of poor-outcome therapists in comparison to good-outcome therapists;
b) A very high consistency in the dynamics of both therapists and patients (average Pearson *r* = 0.78; st. dev. = 0.46 and *r* = 0.82; st. dev. = 0.10 for therapists and patients, respectively), as well as in the way they interacted (Patient-to-Therapist symbolic dynamics, average Pearson correlation: *r* = 0.872; st. dev. = 0.038; Therapist-to-Patient symbolic dynamics, average Pearson correlation: *r* = 0.848; st. dev. = 0.044; between Patient-to-Therapist and Therapist-to-Patient symbolic dynamics, average Pearson correlation: *r* = 0.892; st. dev. = 0.032) was found.

The first observation (a) can be traced back to different findings:

– The results of “static analyses”, as presented in the previous paper resulting from this study (de Felice et al., 2019b): when patients made use of abstract language, they were interpreted very positively by poor-outcome therapists but very negatively by good-outcome therapists. The good-outcome therapists probably (and correctly) considered this behavior as a patient’s defense mechanism that needs to be addressed, while poor-outcome therapists considered this as a sign of working through.
– The discrepancy (DeltaCorr, Table 3) in the therapists and patients’ dynamics proved to be two times higher within poor–poor correlations than in good–good correlations, and of opposite sign (mean DeltaCorr, poor–poor = 0.105 vs. good–good = −0.048, Table 3).
– In the multiple regression models, the composite frequency weighed 2.4 and 17 times more than the Euclidean distance for patients and therapists, respectively. The difference was mainly explained by their β distance coefficients: the therapists’ transitions depended approximately 10 times less on distance than those of patients (ratio between absolute values of their β coefficients = 0.343/0.035 = 9.8, Table 5).

Overall, the greater variability in the behavior of poor-outcome therapists reflected their bigger effort to deal with their poor-outcome patients. Nevertheless, it is difficult to say with certainty if this greater variability in their behaviors depended on a deliberately different therapeutic approach or rather due to the difficulty inherent to the clinical process. The differences observed in the correlation matrix between good-outcome and poor-outcome cases seem to support the latter hypothesis. In fact, only the poor-outcome patients made use of positive and negative emotional languages inversely proportional to abstraction (“static analyses”, de Felice et al., 2019b).

The second result of our study (b) represents a prominent proof-of-concept of the possibility to consider psychotherapy as a proper dynamical system, advocating for the application of classical physics-inspired methods to the study of psychotherapy. Even when considering the singularities of each psychotherapeutic relationship, the results of this study demonstrated the existence of a nucleus of invariants amenable to the principles of dynamical systems. The principles ruling the process of patients and therapists’ dynamics were studied by means of multiple regression models that were able to accurately predict their symbolic dynamics by considering the joint frequencies of state *i* and *j* and their distance (average *p* value, patients < 0.0001, therapists < 0.001; average adjusted *R*^2^, patients = 0.774, therapists = 0.684). The results show that their respective systems were not completely ergodic because their transitions depended also on the states in which the transitions started. In other words, their systems’ initial positions influenced the following steps.

As mentioned earlier, this functioning can be described as the “Drunkard’s walk” or “random walk”; in Pearson’s words: “the lesson of Lord Rayleigh’s solution is that in open country the most probable place to find a drunken man who is at all capable of keeping on his feet is somewhere near his starting point!” (1905; p. 294). This statement, from a clinical perspective, could resemble a definition of the Freudian concept of “compulsion to repeat” (Freud, 1914), by which the patient is unconsciously forced to re-experience a traumatic event or a relational pathological attitude while attempting to master the anxiety it provokes. The random-walk-like behavior of the eight psychotherapeutic dyads investigated in this study reflects this mode of functioning on a relational level. The psychotherapeutic interactions moved between adjacent and most occurring linguistic (i.e., mental) states, avoiding transitions toward the very far and least occurring ones (i.e., unexplored mental states). While the good-outcome therapists presumably judged their patients’ networks as functional, the poor-outcome therapists tended to force the psychotherapeutic field toward the functional states more eagerly. This would explain the greater variability in the linguistic behavior of poor-outcome therapists. The clinical nucleus on which their concerns are focused seems to be related to the rationalization dynamic that emerged by means of “static analyses” (de Felice et al., 2019b). Only the poor-outcome patients made a use of positive and negative emotional language inversely proportional to abstraction, which suggests that they probably used the clinical setting to speak about concrete issues while avoiding emotional involvement. An open question concerns the poor-outcome therapists’ awareness of that rationalization dynamic or, conversely, their limited ability to address it.

Despite the remaining questions, the methodology introduced in the present paper has the potential to open new avenues and to raise and answer new questions in psychotherapy research. Just to mention some of them, further research could study the following: the time spent in a dysfunctional state in poor- and good-outcome dyads, the minimum number of oscillations to produce an entirely new state or attractor, the way a therapist’s intervention impacts the patient’s network, the differences between networks of diverse psychotherapeutic approaches, and the treatment outcomes in relation to specific transition dynamics. All these research questions can be investigated by means of symbolic dynamics, using psychophysiological variables, such as heartbeat or galvanic skin response, as well as linguistic and non-verbal variables (e.g., Giuliani et al., 1994; Gorban et al., 2010; Halfon et al., 2016; Orsucci et al., 2016; Rybnikov et al., 2017). Moreover, other than the Euclidean, in future research, a fruitful investigation could concern the use of different distances between clusters, such as Manhattan and Mahalanobis, to analyze specific psychotherapeutic networks and clinical dynamics. In summary, studying psychotherapy in terms of complex systems and visualizing the psychotherapeutic field (Baranger and Baranger, 1961) as a network allows us to investigate psychotherapeutic evolutions over time and process-outcome relations in a completely novel and data-driven manner. The methodology presented in this manuscript can foster further efforts in the line of research that aims to unite psychotherapy and complexity science (de Felice et al., 2019a). Although we are perfectly aware that the analysis of eight brief psychotherapies poses severe limitations to the generalizability of our results, we are profoundly convinced that the importance and innovation of methods can represent a generative substratum capable of overshadowing that criticality.

Finally, we return to the two generic research questions that guided the research project (presented in the current and previous paper, see de Felice et al., 2019b): (a) the possibility of predicting good- and poor-outcome psychotherapies on the sole basis of the correlation pattern among their macro-parameters, and (b) the investigation, in terms of those correlation patterns, of analogies and differences between the processes of good- and poor-outcome cases.

a) Regarding the first question, our analyses confirmed the possibility to predict good- and poor-outcome psychotherapies on the sole basis of the correlation patterns among their macro-parameters. The results of the “dynamic analyses” presented in the previous paper (de Felice et al., 2019b) demonstrated the statistical significance of five macro-parameters, grouped into two main dimensions of order-variability and elementary-complex, in describing the singularities of each of the eight psychotherapeutic processes. Hence, we conclude that the rationale of Statistical Mechanics, which uses probability theory to study and predict the average behavior of systems in which microscopic details are obscure and/or not measurable, proved to be not only suitable but also fundamental in producing a significant advancement in the psychotherapy research literature (de Felice et al., 2019b).
b) The second question was addressed in the present study. Analogies between the processes of good- and poor-outcome cases were demonstrated by the high consistency of the patients’ dynamics, the therapists’ dynamics and their interactions (Patient-to-Therapist and Therapist-to-Patient dynamics). Furthermore, by studying the principles ruling these dynamics, it was possible to observe their random-walk-like behavior. As such, the mind seems to be tied to its initial position or the “ordinary mental state.” Subsequently, it is able to move toward diverse mental states, which, under the influence of some kind of homeostasis principle, appear to be the more adjacent and most occurring ones. Sigmund Freud called the tendency to repeat familiar, even if traumatic mental states, while avoiding the unexplored ones, “compulsion to repeat” (Freud, 1914). Even the most recent psychoanalytic theories, albeit with some differences, agree on the idea that patients use rigid relational patterns resulting from past relational experiences (e.g., Mitchell, 1993, 2014; Bromberg, 1998; Stern, 2013; Hoffman, 2014). Psychopathology, as well as psychic suffering, is therefore currently understood as the tendency to rigidly reiterate relational dysfunctional patterns and, consequently, therapeutic change is conceived as the gradual shift from rigid and repetitive relational patterns to more flexible ones (Bromberg, 2001; Stern, 2001).

On the other hand, the differences in the processes of good- and poor-outcome cases, as observed in this study, resulted from the greater variability in the behavior of poor-outcome therapists and the inversely proportional use of positive/negative emotional language and abstraction in poor-outcome patients. This result has been interpreted as a dynamic of rationalization characterizing the poor-outcome dyads.

In contrast to mainstream psychotherapy research characterized by the endless search for increasingly detailed mediation, moderation, hierarchical, multilevel models to explain the outcome of psychotherapy, the present study establishes the strength of a scientific effort that follows a completely different route: abstracting significant *trans-*theoretical, data-driven macro-parameters and studying their interactions over time. After confirming the possibility of collapsing eight psychotherapeutic processes into two main dimensions (order-variability and elementary-complex, see part one: de Felice et al., 2019b), the present study not only showed how to describe the clinical interactions in terms of networks but also found out the main principles on which their evolutions were based. The mind, from a linguistic perspective, seems to work-through psychotherapy sessions by passing from the most adjacent states and the most occurring ones. This finding can represent a fertile ground to rethink pivotal clinical concepts such as the timing of an interpretation or a comment, the clinical issue to address within a given session and the general task of a psychotherapist: from someone who delivers a given technique toward a consultant promoting the flexibility of the clinical field and, thus, of the patient’s mind. Hence, we can recommend that the clinician should promote the patient’s passage toward less explored mental states by softening the degree of anxiety they convey. In so doing, the patient’s personality is enriched and he/she acquires the capacity of “feeling, thinking and being” (Matte-Blanco, 1988) previously unfamiliar internal aspects (anxiety-triggering). By this process, the patient will gain not only internal freedom but also the capacity of doing new experiences, different from the old relational pattern. Therefore, in terms of psychotherapeutic training, we should foster the competence of clinicians to *observe* the network of the patient’s mind as-a-whole; to *listen to* the patient’s need of keeping his/her mind within a certain dysfunctional organization (attractor) together with his/her desire to change (i.e., understanding what are the mental states impossible to integrate because of the anxiety they convey); to *interpret*, that is, promoting the emergence, in the patient’s mind, of a more functional state or group of states (attractor) in which he/she can reside regardless of the anxiety they can, especially at the beginning, provoke. Note that the word “interpretation”, although much more used in the psychodynamic schools, can be seen as having both behavioral and verbal components. Even an orthodox psychoanalyst is constantly delivering a behavioral treatment: despite the possible hate or any kind of attack the patient can address him/her, he/she continues to be there, listening to his/her patient. Hence, in this context, the word interpretation should be considered as a *trans-*theoretical capacity of the clinician to let emerge, in the clinical relationship, new behavioral and relational patterns, previously unexplored because of the anxiety they triggered. Observation, listening, and interpretation, from this perspective, can be considered as the three main elements of the clinical relationship and training (de Felice, 2020).

## Data Availability Statement

The datasets generated for this study are available on request to the corresponding author.

## Author Contributions

GF and AG: conceptualization. GF, AG, AS, and FO: methodology. GF, AG, AS, OG, and EM: formal analysis. GP, MD, and SA: data curation. GF, FO, GS, and RM: writing – original draft preparation. GF, AG, MD, OG, SA, GP, GS and RM: writing, review, and editing.

## Conflict of Interest

The authors declare that the research was conducted in the absence of any commercial or financial relationships that could be construed as a potential conflict of interest.
