# Supplementary material for: What Differentiates Poor- and Good-Outcome Psychotherapy? A Statistical-Mechanics-Inspired Approach to Psychotherapy Research, Part Two: Network Analyses
Source: Front Psychol. 2020 May 20;11:788. doi: 10.3389/fpsyg.2020.00788 (PMC7251305; doi:10.3389/fpsyg.2020.00788)
Supplement: Supplementary file 1 [file Data_Sheet_1.docx]

**Appendix 1.** **Summary of the symbolic dynamic procedure.**

**Table I.** Summary of the symbolic dynamic procedure.

| **Summary of the symbolic dynamic procedure** | | | | | | | | |
| --- | --- | --- | --- | --- | --- | --- | --- | --- |
| *Statistical Unit* | *Abstract Language, Patient* | *Positive Emotional Language, Patient* | *Negative Emotional Language, Patient* | *Symbolic Dynamic of Patients* | *Abstract Language, Therapist* | *Positive Emotional Language, Therapist* | *Negative Emotional Language, Therapist* | *Symbolic Dynamic of Therapists* |
| Patient George, from word 1 to 150 | AB relative frequency | POS relative frequency | NEG relative frequency | number of cluster from 1 to 8 | AB relative frequency | POS relative frequency | NEG relative frequency | number of cluster from 1 to 8 |
| from word 151 to 300 | … | … | … | … | … | … | … | … |
| from word 301 to 450 | … | … | … | … | … | … | … | … |
| from word (n-150) to (n) | … | … | … | the sequence of clusters (discrete combinations of the three vocabularies) is the symbolic dynamic of patients and can be transformed into a network by means of Markov matrices | … | … | … | the sequence of clusters (discrete combinations of the three vocabularies) is the symbolic dynamic of therapists and can be transformed into a network by means of Markov matrices |

**Appendix 2: Multidimensional scaling planes, clusters’ frequencies and centroids of the patients’ and therapists’ phase space.**

Multidimensional scaling attempts to find the structure in a set of distance measures between objects or cases. This task is accomplished by assigning observations to specific locations in a conceptual space (usually two- or three-dimensional); the distances between the points in the space match the given dissimilarities as closely as possible. In many cases, the dimensions of this conceptual space can be interpreted and used to further understand the data.

*Figure I. The patients’ phase space before and after “outliers” removal.*


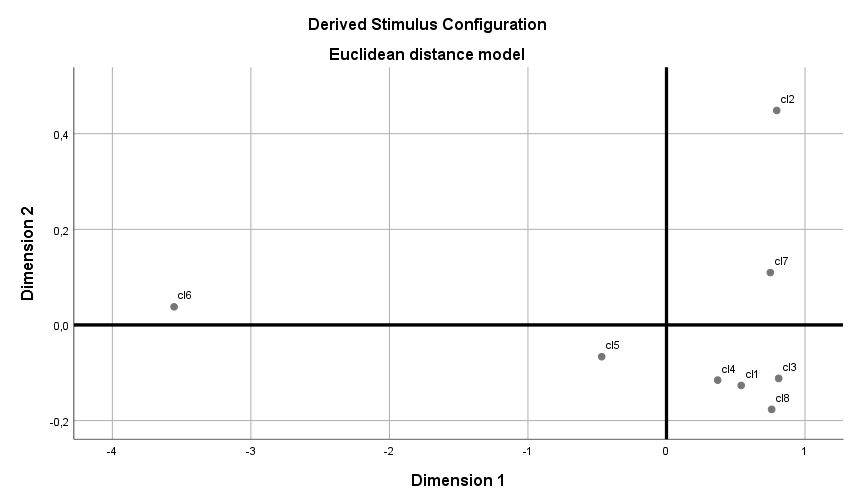

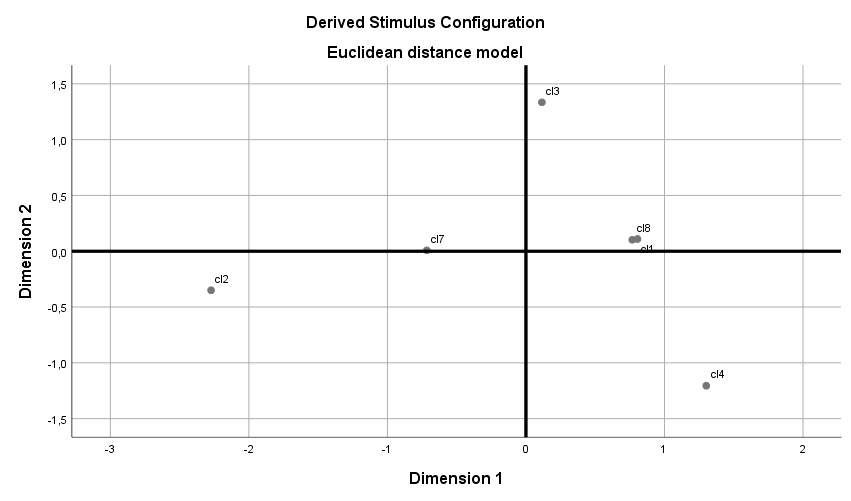


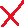

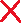


**Table II.** Patients. Clusters’ centroids. The values are standardised (mean=0 and st.dev.=1). The highest absolute values for clusters located at the poles of the plane are indicated in bold. It is worth noting the different pattern of signs (+/-) for each cluster.

| **Clusters’ Centroids** | | | | | | | | | |
| --- | --- | --- | --- | --- | --- | --- | --- | --- | --- |
|  | Cluster | | | | | | | | |
|  | 1 | 2 | 3 | 4 | 5 | 6 | 7 | 8 |  |
| R-AB patient | 0.637 | 0.098 | **1.173** | -0.718 |  |  | -0.301 | -0.695 |  |
| R-POS patient | 0.833 | -0.315 | -0.503 | **1.582** |  |  | -0.247 | -0.444 |  |
| R-NEG patient | -0.396 | **2.656** | -0.213 | -0.409 |  |  | 0.895 | -0.571 |  |

**Table III.** Patients. Clusters’ frequencies.

| **Clusters’ Frequencies** | | |
| --- | --- | --- |
| Cluster | 1 | 1115 |
|  | 2 | 371 |
|  | 3 | 1494 |
|  | 4 | 709 |
|  | ~~5~~ | ~~23~~ |
|  | ~~6~~ | ~~1~~ |
|  | 7 | 1484 |
|  | 8 | 2191 |
| Valid | | 7388 |
| Missing | | 0 |

*Figure II. The therapists’ phase space before and after “outliers” removal.*


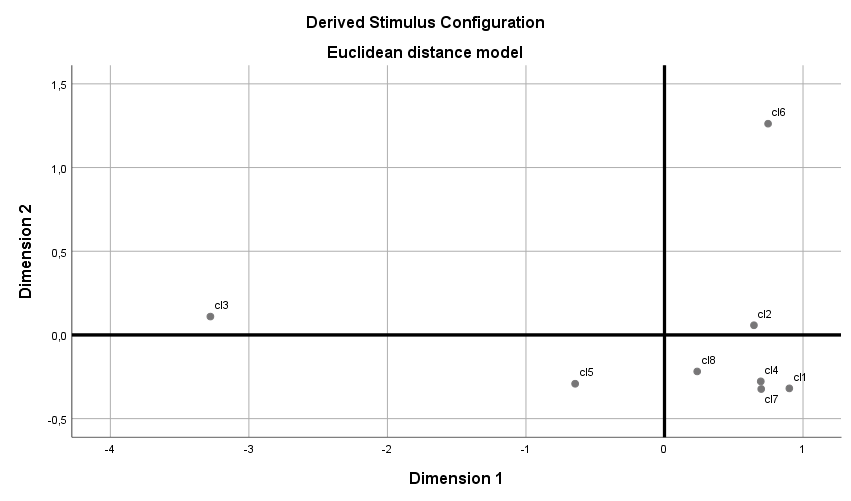

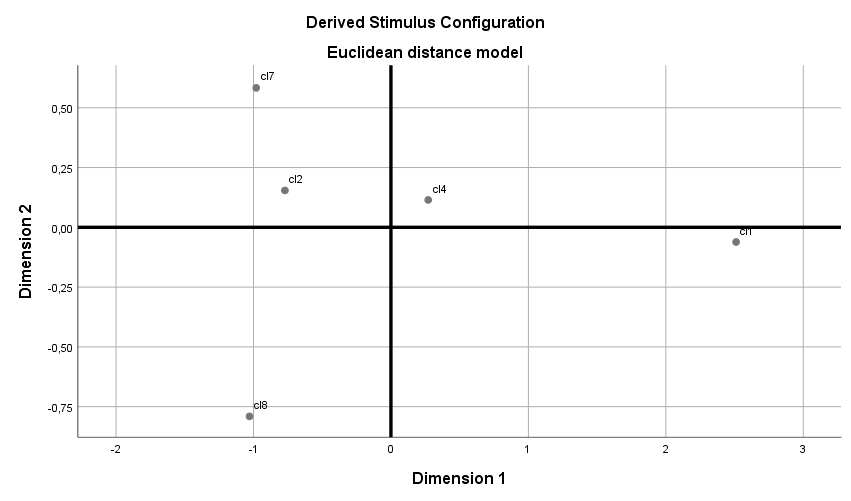


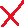

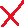

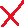


**Table IV.** Therapists. Clusters’ centroids. The values are standardised (mean=0 and st.dev.=1). The highest absolute values for clusters located at the poles of the plane are indicated in bold. It is worth noting the different pattern of signs (+/-) for each cluster.

| **Clusters’ Centroids** | | | | | | | | | |
| --- | --- | --- | --- | --- | --- | --- | --- | --- | --- |
|  | Cluster | | | | | | | | |
|  | 1 | 2 | 3 | 4 | 5 | 6 | 7 | 8 |  |
| R-AB therapist | 0.324 | 0.935 |  | 0.011 |  |  | **-0.712** | -0.053 |  |
| R-POS therapist | -0.566 | -0.194 |  | -0.234 |  |  | -0.450 | **1.318** |  |
| R-NEG therapist | **4.132** | -0.285 |  | 1.183 |  |  | -0.494 | -0.268 |  |

**Table V.** Therapists. Clusters’ frequencies.


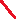


| **Clusters’ Frequencies** | | |
| --- | --- | --- |
| Cluster | 1 | 138 |
|  | 2 | 1860 |
|  | ~~3~~ | ~~3~~ |
|  | 4 | 1393 |
|  | ~~5~~ | ~~73~~ |
|  | ~~6~~ | ~~35~~ |
|  | 7 | 2682 |
|  | 8 | 1204 |
| Valid | | 7388 |
| Missing | | 0 |

Observing the multidimensional scaling planes, it is evident, in both patients and therapists, that the first dimension is pulled by negative language (at the left and right pole respectively). The second dimension, on the other hand, has positive emotional language and abstract language as poles, however, while both the vocabularies have a positive sign for the patients, they show opposite signs in the case of the therapists. Taken together, these distinctions reflect the different evaluations of abstraction made by the therapists in poor and good outcome cases: as mentioned in the introduction, the “static analyses” (see de Felice et al., 2019b) suggested that only the therapists of good outcome cases seem to consider abstraction as a defence mechanism against the emotional involvement required in a therapeutic process. The therapists of poor outcome cases on the other hand, seem to consider the patient’s use of abstract language as a positive sign of working through.
